# Supplementary material for: Predictive modeling of apple slice drying: Integrating temperature, thickness, and shrinkage dynamics
Source: PLoS One. 2026 Mar 31;21(3):e0341428. doi: 10.1371/journal.pone.0341428 (PMC13038015; doi:10.1371/journal.pone.0341428)
Supplement: Attachment — Submitted filename: Comments on PONE-D-25-36704.docx [file pone.0341428.s017.docx]

**Predictive Modeling of Apple Slice Drying: Integrating Temperature, Thickness, and Shrinkage Dynamics**

The authors have undertaken a rigorous experimental and modeling approach to investigate the complex interplay between drying temperature, apple slice thickness, and the resulting kinetics, shrinkage, and color changes. However, the manuscript currently suffers from a perceived lack of innovation. While the study is technically sound, it does not sufficiently articulate its unique contribution to the field. Many studies have explored the effects of temperature and thickness on apple drying. The authors should work on more clearly showing the innovation of their work compared with existing studies in the literature. Is it the specific combination of models? Highlighting this novelty is critical. While the findings are valuable, their impact is diminished without a clear statement of what this work achieves that previous studies have not. The following detailed comments are provided to assist the authors in strengthening the paper, with a particular focus on better contextualizing its innovative aspects, to elevate it for publication.

**Comment 1:**

On page 11 (line 113), the text states the experiments were conducted in the "summer of 2024." Please correct this expression.

**Comment 2:**

On page 12 (line 146), the text refers to "Equation 5," but the corresponding equation is numbered as (4) (line 155). Please ensure all in-text citations to equations, figures, and tables are accurate.

**Comment 3:**

The Introduction mentions the use of the "Golden Delicious" apple variety but does not explain why this specific cultivar was chosen. Providing this context strengthens the experimental design. Please add a sentence in the Introduction or Materials and Methods section explaining the rationale for selecting Golden Delicious apples and what make this varietyan ideal model for this study.

**Comment 4:**

Please perform an Analysis of Variance (ANOVA) on the total color difference (ΔE) values presented in Figure 14. This will statistically validate whether the differences in color change across the various drying treatments are significant, adding a quantitative layer to your qualitative discussion.

**Comment 5:**

In the discussion of the multivariate regression model for moisture diffusivity (page 22, lines 339-344), the interpretation could be clearer and more direct. Please rephrase the analysis of the T² and T terms to be more intuitive.

**Comment 6:**

The explanation for why thinner slices have higher activation energy (page 23, lines 361-364) is slightly counter-intuitive and could be clarified. The current text states that thinner slices lose moisture quickly, meaning "more energy is needed for moisture to diffuse." Please refine this explanation and clarify the discussion on Activation Energy.

**Comment 7:**

The manuscript briefly mentions the assumption of isotropic shrinkage as a limitation (page 27). This section could be expanded to provide more specific directions for future research. In the final paragraph of the "Statistical Analysis" section or in the "Conclusion," please elaborate on this and suggest specific future work.

**Comment 8:**

Several figures and tables are difficult to read due to small font sizes, which will be a problem in the final publication. Please increase the font size for axis titles, labels, and legends in all graphs, especially Figures 15 and 16. Ensure that all text within tables is legible and that the table titles (e.g., Table 2) clearly describe the contents, including the statistical test used (e.g., "ANOVA results for the effect...").. Consider improving Readability!

**Comment 9:**

Please structure the abstract to clearly separate the key findings from their implications. For instance, explicitly state the main quantitative results and conclude with a clear statement on the optimal conditions found.

**Comment 10:**

Please carefully proofread the entire manuscript to correct minor typos and grammatical errors. For example, on page 9 (line 70), there is a stray "y" at the start of a sentence. A thorough check will ensure the final version is polished.
