# Supplementary material for: Predictive modeling of apple slice drying: Integrating temperature, thickness, and shrinkage dynamics
Source: PLoS One. 2026 Mar 31;21(3):e0341428. doi: 10.1371/journal.pone.0341428 (PMC13038015; doi:10.1371/journal.pone.0341428)
Supplement: Attachment — Submitted filename: Comments- PONE-D-25-36704.docx [file pone.0341428.s018.docx]

**Comments**

The manuscript titled *“Predictive Modeling of Apple Slice Drying: Integrating Temperature, Thickness, and Shrinkage Dynamics”* presents valuable experimental and modeling insights, but several technical clarifications and refinements are needed for strengthening the scientific quality. Below are comments

| **S. No.** | **Section / Page** | **Comment** |
| --- | --- | --- |
| 1. | Abstract (p.1) | The abstract is informative, but it would benefit from explicitly mentioning the experimental replication and statistical approach used for validation. |
| 2. | Abstract (p.1) | The conclusion on “optimal visual quality at higher temperatures” seems counterintuitive; please justify with supporting data or revise the phrasing. |
| 3. | Introduction (p.2) | The introduction provides background, but it should briefly highlight gaps in existing FEM-based drying studies for apples to justify novelty. |
| 4. | Introduction (p.3) | The statement that thickness has a greater effect than temperature needs citations of at least two prior studies to reinforce the claim. |
| 5. | Materials & Methods (Drying System, p.4) | The detailed hardware description (brands, model numbers) is good, but airflow distribution uniformity inside the cabinet should be validated, as it significantly affects results. |
| 6. | Methods (p.5) | Replication strategy (triplicate runs) is mentioned; however, error bars or confidence intervals should be presented in figures to reflect variability. |
| 7. | Theoretical Modeling (p.7) | The assumption of constant diffusivity in FEM needs a stronger justification; real food tissues often show variable diffusivity during drying. |
| 8. | Modeling Equations (p.8) | The convective mass transfer coefficient (hD) correlations are generic; please explain why these are suitable for apple slices and the airflow regime used. |
| 9. | Results (Drying Curves, p.11) | Figures 5–7 lack error bars; inclusion would enhance credibility of experimental reproducibility. |
| 10. | Results (Drying Time Trends, p.12) | The repetition of drying time values in text and figure captions is redundant—consider condensing to avoid over-description. |
| 11. | Shrinkage Analysis (p.13) | Shrinkage is well modeled, but microstructural imaging (SEM or microscopy) would provide mechanistic support for observed trends. |
| 12. | Shrinkage Regression Model (p.14) | The regression equation (Eq. 14) assumes linearity; discussion on possible non-linear relationships should be added. |
| 13. | Moisture Diffusivity (p.15) | The claim that diffusivity increases with thickness contradicts conventional expectations; please discuss physical explanation in more depth. |
| 14. | Activation Energy (p.17) | The activation energy values differ widely between thicknesses; a discussion on structural resistance differences should be expanded. |
| 15. | Colour Analysis (p.18) | Table 4 reports L*, a*, b* values, but ΔE values are not consistently included; integrating them would improve clarity. |
| 16. | Colour Results (p.19) | The reasoning for higher ΔE in thinner slices at 70°C is logical, but experimental replicates should be shown in graphs for robustness. |
| 17. | Modeling Validation (p.20) | Figure 16 shows close agreement between model and experiment, but statistical indices like chi-square or Willmott’s d-index could strengthen validation. |
| 18. | Discussion (p.21) | While FEM assumptions are acknowledged, anisotropic shrinkage modeling should be suggested more prominently as a future direction. |
| 19. | Conclusion (p.22) | The conclusion is comprehensive but could highlight the industrial applicability (e.g., energy savings, product uniformity) more clearly. |

Overall, the study is well-executed and provides significant contributions to predictive drying modeling. Addressing the above comments will improve methodological rigor, enhance clarity, and strengthen the manuscript’s suitability for publication.
