# Supplementary material for: Predictive modeling of apple slice drying: Integrating temperature, thickness, and shrinkage dynamics
Source: PLoS One. 2026 Mar 31;21(3):e0341428. doi: 10.1371/journal.pone.0341428 (PMC13038015; doi:10.1371/journal.pone.0341428)
Supplement: Attachment — Submitted filename: Respond letter.docx [file pone.0341428.s020.docx]

**Dear Editor and Reviewers,**

We sincerely thank you for your careful assessment of our manuscript and for the valuable comments and suggestions provided. We have carefully revised the manuscript in accordance with all recommendations. Below, we provide a detailed, point-by-point response outlining the changes made and clarifying how each comment has been addressed.

We appreciate your time and consideration and believe that the revisions have substantially improved the quality and clarity of our work. Please find our detailed responses below.

**Editor comment:**

We note that Figures 1, 2, and 3 in your submission may contain copyrighted images. All PLOS content is published under the Creative Commons Attribution License (CC BY 4.0), which means that the manuscript, images, and Supporting Information files will be freely available online, and any third party is permitted to access, download, copy, distribute, and use these materials in any way, even commercially, with proper attribution.

Response: Thank you for the notification. We have replaced Figure 1 with an original schematic illustration and have removed Figures 2 and 3 to ensure that all figures comply with CC BY 4.0 licensing requirements.

**Reviewer 1:**

**Predictive Modeling of Apple Slice Drying: Integrating Temperature, Thickness, and Shrinkage Dynamics**

The authors have undertaken a rigorous experimental and modeling approach to investigate the complex interplay between drying temperature, apple slice thickness, and the resulting kinetics, shrinkage, and color changes. However, the manuscript currently suffers from a perceived lack of innovation. While the study is technically sound, it does not sufficiently articulate its unique contribution to the field. Many studies have explored the effects of temperature and thickness on apple drying. The authors should work on more clearly showing the innovation of their work compared with existing studies in the literature. Is it the specific combination of models? Highlighting this novelty is critical. While the findings are valuable, their impact is diminished without a clear statement of what this work achieves that previous studies have not. The following detailed comments are provided to assist the authors in strengthening the paper, with a particular focus on better contextualizing its innovative aspects, to elevate it for publication.

**Response:** Thank you for your suggestion. The Introduction has been revised to clearly highlight the novelty of this work. The revised text emphasizes that this study uniquely integrates experimental measurements with predictive modeling, combining multivariate regression and Finite Element Modeling (FEM) to simultaneously quantify drying kinetics, shrinkage, moisture diffusivity, and color changes. Predictive models were developed to express shrinkage and moisture diffusivity explicitly as functions of slice thickness and temperature, providing a reliable, quantitative tool for optimizing drying parameters. This integrated approach enables accurate prediction of both physical and quality-related changes under varying conditions, offering actionable guidance for designing energy-efficient drying systems—a contribution that has not been addressed in previous studies.

**Comment 1:**

On page 11 (line 113), the text states the experiments were conducted in the "summer of 2024." Please correct this expression.

**Response:** Thank you for your comment. The experiments were indeed performed in June 2024, before the manuscript was submitted. The sentence has been revised to: ‘After setting up the system, the drying experiments were carried out in June 2024 at the Department of Biosystems Mechanical Engineering, Shiraz University.

**Comment 2:**

On page 12 (line 146), the text refers to "Equation 5," but the corresponding equation is numbered as (4) (line 155). Please ensure all in-text citations to equations, figures, and tables are accurate.

**Response:** Thank you for your comment. All equation, figure, and table numbers have been checked and updated as appropriate.

**Comment 3:**

The Introduction mentions the use of the "Golden Delicious" apple variety but does not explain why this specific cultivar was chosen. Providing this context strengthens the experimental design. Please add a sentence in the Introduction or Materials and Methods section explaining the rationale for selecting Golden Delicious apples and what make this varietyan ideal model for this study.

**Response:** Thank you for your comment. A sentence has been added to the Materials and Methods explaining the rationale for selecting the ‘Golden Delicious’ cultivar.

“The Golden Delicious apple was chosen due to its global commercial relevance, uniform morphology, and stable chemical composition, which minimize variability and make it a suitable model cultivar for evaluating drying performance and quality changes.”

**Comment 4:**

Please perform an Analysis of Variance (ANOVA) on the total color difference (ΔE) values presented in Figure 14. This will statistically validate whether the differences in color change across the various drying treatments are significant, adding a quantitative layer to your qualitative discussion.

**Response:** Thank you for your suggestion. An ANOVA was conducted to evaluate the effects of drying temperature, slice thickness, and their interaction on the total color difference (ΔE). The analysis revealed that temperature, thickness, and their interaction all significantly affected ΔE (p < 0.0001). These results have been included in Table 4 and discussed in the revised manuscript to provide a quantitative assessment of color change across the drying treatments.

**Comment 5:**

In the discussion of the multivariate regression model for moisture diffusivity (page 22, lines 339-344), the interpretation could be clearer and more direct. Please rephrase the analysis of the T² and T terms to be more intuitive.

**Response:** Thank you for this suggestion. The paragraph discussing the regression model has been revised to provide a clearer and more intuitive interpretation of the temperature terms (T² and T). The updated text explains that these terms together describe a non-linear effect of temperature on moisture diffusivity, with Deff generally increasing at higher temperatures. Slice thickness effects have also been clarified, emphasizing that thicker slices lead to higher Deff. The revised paragraph improves readability while maintaining the technical accuracy of the model.

**Comment 6:**

The explanation for why thinner slices have higher activation energy (page 23, lines 361-364) is slightly counter-intuitive and could be clarified. The current text states that thinner slices lose moisture quickly, meaning "more energy is needed for moisture to diffuse." Please refine this explanation and clarify the discussion on Activation Energy.

**Response:** Thank you for this valuable comment. The paragraph discussing activation energy has been revised to provide a clearer and more intuitive explanation. The revised text clarifies that thinner slices have a larger surface-to-volume ratio, which leads to faster initial moisture loss at the surface. As a result, maintaining effective moisture transport in thinner slices requires more energy, which is reflected in their higher activation energy values. In contrast, thicker slices dry more slowly and uniformly, making their moisture diffusion less sensitive to temperature changes. The revised paragraph also highlights supporting findings from previous studies to contextualize these observations.

**Comment 7:**

The manuscript briefly mentions the assumption of isotropic shrinkage as a limitation (page 27). This section could be expanded to provide more specific directions for future research. In the final paragraph of the "Statistical Analysis" section or in the "Conclusion," please elaborate on this and suggest specific future work.

**Response:** Thank you for your suggestion. The Conclusion has been revised to provide a more detailed discussion of the limitations related to isotropic shrinkage and directions for future research. The updated text now highlights the potential for incorporating anisotropic shrinkage effects and variable tissue properties, extending the model to three dimensions, and performing experimental validation using non-destructive imaging or real-time moisture sensors. These additions provide specific and actionable directions for future studies and enhance the clarity and applicability of the conclusions.

**Comment 8:**

Several figures and tables are difficult to read due to small font sizes, which will be a problem in the final publication. Please increase the font size for axis titles, labels, and legends in all graphs, especially Figures 15 and 16. Ensure that all text within tables is legible and that the table titles (e.g., Table 2) clearly describe the contents, including the statistical test used (e.g., "ANOVA results for the effect...").. Consider improving Readability!

**Response:** Thank you. The font size of the figures has been increased, and the titles of the related tables have been revised.

**Comment 9:**

Please structure the abstract to clearly separate the key findings from their implications. For instance, explicitly state the main quantitative results and conclude with a clear statement on the optimal conditions found.

**Response:** Thank you for the suggestion. The abstract has been revised to clearly highlight the key findings and their implications in a single, coherent paragraph. Quantitative results such as the effects of temperature and slice thickness on drying time, shrinkage, and moisture diffusivity are explicitly stated. The optimal drying conditions (70°C and 4 mm slice thickness) are also clearly indicated, along with recommendations for future research, including anisotropic shrinkage modeling, energy analysis, and hybrid drying techniques. This revision improves clarity and ensures that both the main results and their significance are immediately understandable to the reader.

**Comment 10:**

Please carefully proofread the entire manuscript to correct minor typos and grammatical errors. For example, on page 9 (line 70), there is a stray "y" at the start of a sentence. A thorough check will ensure the final version is polished.

**Response:**
Thank you for pointing this out. The entire manuscript has been thoroughly proofread, and all identified typographical and grammatical errors, including the stray “y” on page 9, line 70, have been corrected. Additional revisions were made to improve clarity, readability, and consistency throughout the text.

Reviewer 2

**Comment 1**

The abstract is informative, but it would benefit from explicitly mentioning the experimental replication and statistical approach used for validation.

**Response:**

Thank you for the helpful suggestion. The abstract has been revised to include a clear statement that all drying experiments were performed in triplicate and that statistical validation, including Analysis of Variance (ANOVA), was used to assess the significance of the effects of temperature and slice thickness. This addition strengthens the methodological clarity of the abstract and highlights the rigor of the study’s experimental and analytical approach.

**Commnet2:**

The conclusion on “optimal visual quality at higher temperatures” seems counterintuitive; please justify with supporting data or revise the phrasing.

**Response:** Thank you for this observation. We agree that stating “optimal visual quality at higher temperatures” in the abstract may appear counterintuitive without context. We have revised the abstract to clarify that the improved visual quality was specifically associated with the *combination* of 70°C and a 4 mm slice thickness, which resulted in the lowest color change (ΔE) among all treatments. This combination reduced overall drying time enough to limit enzymatic browning, despite the high temperature. The revised phrasing now clearly reflects this interaction and avoids any misleading generalization about high-temperature drying.

**Comment3:** The introduction provides background, but it should briefly highlight gaps in existing FEM-based drying studies for apples to justify novelty.

**Response:** Thanks for your comment. In this regard, we developed the last section of introduction to highlight the novelty of the research, in use of FEM to simultaneously quantify multiple aspects of the drying process. The revised section is as following:

“This study investigates the effects of slice thickness and drying temperature on the drying kinetics, moisture diffusivity, shrinkage, and color of apple slices. A key innovation of this work lies in the **integration of experimental measurements with predictive modeling**, combining multivariate regression and Finite Element Modeling (FEM) to simultaneously quantify multiple aspects of the drying process. Predictive models were developed to express shrinkage and moisture diffusivity explicitly as functions of slice thickness and temperature, providing a **reliable, quantitative tool** for optimizing drying parameters and improving both efficiency and product quality. Unlike previous studies, which often focus on individual factors or qualitative trends, this integrated approach enables accurate prediction of physical and quality-related changes under varying operational conditions, offering actionable insights for designing energy-efficient drying systems tailored to specific product characteristics.”

**Comment4:** The statement that thickness has a greater effect than temperature needs citations of at least two prior studies to reinforce the claim.

**Response:** Thank you for this helpful comment. We have now added supporting literature that directly demonstrates the comparatively stronger influence of slice thickness on drying time. Studies on melon and G. erubescens fruits show that increasing slice thickness results in a substantially greater increase in drying duration than the reductions achieved by increasing temperature. In this regard, following paragraph was added: “Evidence from previous drying studies supports the greater influence of slice thickness compared to temperature on drying time. For instance, in the drying of melon slices, increasing thickness from 3 mm to 5 mm led to increases of 38–87% in drying time across temperatures of 60, 70, 80, and 90°C, whereas raising the air temperature within a constant thickness reduced drying time by only 40–53% [18]. Similarly, in studies on other fruit matrices such as G. erubescens, thicker slices consistently required substantially longer drying periods due to the increased moisture diffusion path [19]. These findings collectively demonstrate that slice thickness exerts a stronger limiting effect on moisture removal than temperature, reinforcing our observation that thickness has a more pronounced impact on drying time.”

**Comment 5**

The detailed hardware description (brands, model numbers) is good, but airflow distribution uniformity inside the cabinet should be validated, as it significantly affects results.

**Response:** We appreciate this valuable observation. In response, we have added a clarification in the Materials and Methods section describing how airflow uniformity was checked prior to experiments. Specifically, airflow velocity was measured at multiple points across the tray area using an anemometer to confirm spatial uniformity. These measurements confirmed that the airflow variation across the drying chamber remained within ±5%, indicating sufficient uniform airflow for reliable drying experiments. This information has been incorporated into the revised manuscript to address the reviewer’s concern.

**Comment6**

Replication strategy (triplicate runs) is mentioned; however, error bars or confidence intervals should be presented in figures to reflect variability.

Response: Thank you. Error bars have been added to the figures.

**Comment7**

The assumption of constant diffusivity in FEM needs a stronger justification; real food tissues often show variable diffusivity during drying.

**Response:**

Thank you for this valuable comment. We would like to clarify that the FEM simulations in this study **did not** assume constant moisture diffusivity. The discussion of constant diffusivity in the theoretical modeling section was included only to explain why analytical solutions traditionally rely on that assumption. In our work, however, the effective moisture diffusivity was treated as **variable**, using experimentally determined values for each drying condition to better represent the real behavior of apple tissue during drying. As shown in Equation (16) in the Results and Discussion section, the diffusivity is modeled as a function of both temperature and slice thickness. The theoretical modeling section has now been revised to clearly emphasize this point and eliminate any potential ambiguity.

**Comment8**

The convective mass transfer coefficient (hD) correlations are generic; please explain why these are suitable for apple slices and the airflow regime used.

**Response:**

Thank you for this important point. We have added two references in which the Sherwood relation was used to estimate the convective mass-transfer coefficient for flat-plate materials, such as the apple slices used in our study (Equations 7–10). The added paragraph is as follows:

“These correlations were selected because the airflow in the drying chamber produced Reynolds numbers below 3 × 10⁵, which fall within the laminar–transitional regime where Sherwood–Reynolds–Schmidt correlations are applicable. Apple slices are thin, smooth, and approximately flat, making their geometry compatible with the flat‑plate assumptions underlying these correlations. Such correlations have been successfully applied in previous drying studies of fruits and vegetables with similar geometries and airflow conditions, supporting their suitability for estimating the convective mass-transfer coefficient (hD) in this study. For example, Tuly et al. [16] calculated the mass-transfer coefficient using the Sherwood number (Sh) and Schmidt number (Sc) as given in Equations (7–10). Additionally, Kumar et al. [17] developed a multiphysics drying model in which mass transfer was solved numerically with variable properties, employing Sherwood–Reynolds–Schmidt type correlations.”

**Comment 9**

Figures 5–7 lack error bars; inclusion would enhance credibility of experimental reproducibility

**Response:** Thank you. Error bars have been added to the figures.

**Comment 10**

The repetition of drying time values in text and figure captions is redundant—consider condensing to avoid over-description.

Response: Thanks. the paragraph revised as following to remove repetition of drying time values at text:

Drying time decreased with increasing temperature and decreasing slice thickness, with thicker slices requiring substantially longer times than thinner ones. At 50°C, 60°C, and 70°C, the trends are consistent across all thicknesses, as illustrated in Figure 6. Higher temperatures effectively mitigate the impact of slice thickness on drying time, especially for thicker slices.

**Comment 11**

Shrinkage is well modeled, but microstructural imaging (SEM or microscopy) would provide mechanistic support for observed trends.

**Response:**

We thank the reviewer for this valuable suggestion. While we agree that microstructural imaging (e.g., SEM or optical microscopy) can provide mechanistic insights into shrinkage behavior, this study focused on quantitative modeling of shrinkage using experimental measurements and predictive FEM and regression models. The high agreement between the predicted and experimental shrinkage values (R² > 0.97) demonstrates that the models effectively capture the overall shrinkage trends under varying drying temperatures and slice thicknesses.

Due to limitations in equipment, microstructural imaging was not performed. We have added a statement in the manuscript acknowledging this limitation and suggesting that future work could incorporate SEM or other microscopic techniques to provide additional mechanistic validation of the shrinkage behavior.

**Comment 12**

The regression equation (Eq. 14) assumes linearity; discussion on possible non-linear relationships should be added.

**Response:**

We thank the reviewer for this insightful comment. The regression model presented in Equation (15) was developed based on the observed experimental data, which exhibited an approximately linear relationship between shrinkage, slice thickness, and drying temperature within the studied ranges. While the model assumes linearity, we acknowledge that in broader ranges of temperature or slice thickness, shrinkage behavior may exhibit non-linear trends due to factors such as structural collapse, case hardening, or moisture-dependent diffusivity. In this regard following discussion was added to the text:

“It is important to note that this model assumes a linear relationship; shrinkage may exhibit non-linear behavior at wider ranges of temperature or slice thickness due to phenomena such as structural collapse or moisture-dependent diffusivity”

**Comment 13**

The claim that diffusivity increases with thickness contradicts conventional expectations; please discuss physical explanation in more depth.

**Response:**

We appreciate the reviewer’s observation regarding the apparent counterintuitive trend of increasing diffusivity with slice thickness. While classical theory often suggests that thinner slices facilitate faster moisture transport due to shorter diffusion paths, our results show that effective moisture diffusivity (Deff) appears higher in thicker slices under the studied drying conditions. This can be explained by the larger internal moisture gradients in thicker slices, which create a stronger driving force for moisture diffusion. Additionally, thicker slices may experience slower surface drying initially, maintaining higher internal moisture and enhancing the average effective diffusivity over the drying period.

We have clarified this in the revised manuscript and referenced similar observations in prior studies, such as Royen et al. [5] for apple slices and Limpaiboon [30] for pumpkin slices, to show that this behavior is not unique to our study and can arise under certain drying conditions.

“Although it may seem counterintuitive, effective moisture diffusivity increased with slice thickness due to larger internal moisture gradients in thicker slices, which enhance the driving force for moisture movement. This trend reflects average diffusivity over the drying period rather than local instantaneous values. In a study Royen *et al* [5], moisture diffusivity in apple slices increased with slice thickness, ranging from 4 to 12 mm. Similarly, Limpaiboon [30]. reported that increasing slice thickness and temperature enhanced the moisture diffusivity of pumpkin slices. “

**Comment 14**

The activation energy values differ widely between thicknesses; a discussion on structural resistance differences should be expanded.

**Response:** Thank you for this thoughtful comment. We have expanded the discussion to clarify why activation energy differs across slice thicknesses. The higher activation energy observed in thinner slices is attributed to their greater surface-to-volume ratio and the rapid formation of surface drying fronts, which increase structural resistance to internal moisture movement. Maintaining diffusion under these conditions requires more energy. Thicker slices experience more uniform internal drying and lower structural resistance, making their diffusivity less temperature-dependent and therefore yielding lower activation energy. This explanation has now been added to the manuscript, along with supporting literature (e.g., carrot pomace drying [31]).

**Comment 15**

Table 4 reports L*, a*, b* values, but ΔE values are not consistently included; integrating them would improve clarity.

Response: Thank you. The ΔE values have been included in the table accordingly.

**Comment 16**

The reasoning for higher ΔE in thinner slices at 70°C is logical, but experimental replicates should be shown in graphs for robustness.

**Response:**

Thank you for this valuable comment. We agree that including experimental replicates strengthens the robustness of the results. The variability between replicates was low, and this consistency is reflected in the small standard errors of the fitted models.

Although individual replicate curves were not shown separately in the graphs to maintain clarity, we have now added error bars (representing the standard deviation between replicates) in the corresponding figures. This revision enhances transparency and demonstrates the repeatability of the ΔE measurements. A note has also been added in the Methods section to explicitly state the number of replicates performed for each condition.

**Comment 17**

Figure 16 shows close agreement between model and experiment, but statistical indices like chi-square or Willmott’s d-index could strengthen validation.

**Response:** Thank you for your suggestion. The agreement between model predictions and experimental data in Figure 14 (in new revision, no. of figure is 14) is already quantitatively supported by the statistical indices reported in Table 6, including R², RMSE, and MAE. These metrics indicate a high degree of correlation and low prediction error, providing robust validation of the model. Therefore, the inclusion of chi-square or Willmott’s d-index, while possible, would be complementary, and the current statistical indicators are sufficient to demonstrate model accuracy.

**Comment 18**

While FEM assumptions are acknowledged, anisotropic shrinkage modeling should be suggested more prominently as a future direction.

**Response:**

We thank the reviewer for this suggestion. In the revised manuscript, we have emphasized the need for incorporating anisotropic shrinkage in future studies. Specifically, in the Conclusion, we now highlight that extending the FEM framework to account for anisotropic shrinkage and variable tissue properties would provide a more accurate representation of drying behavior, particularly for thicker apple slices, and could further improve the predictive capability of the model.

**Comment 19**

The conclusion is comprehensive but could highlight the industrial applicability (e.g., energy savings, product uniformity) more clearly.

**Response:**

We thank the reviewer for this valuable suggestion. The conclusion has been revised to explicitly highlight the industrial applicability of our findings. In particular, we emphasized that the results provide actionable insights for designing energy-efficient drying systems that reduce processing time, ensure uniform product quality, and minimize shrinkage-related losses. These aspects underline the practical relevance of our work for industrial-scale apple drying operations.
