# Supplementary material for: Predictive modeling of apple slice drying: Integrating temperature, thickness, and shrinkage dynamics
Source: PLoS One. 2026 Mar 31;21(3):e0341428. doi: 10.1371/journal.pone.0341428 (PMC13038015; doi:10.1371/journal.pone.0341428)
Supplement: Attachment — Submitted filename: Response to Reviewers.docx [file pone.0341428.s021.docx]

**Response to Reviewers**

We sincerely thank the reviewers for their careful evaluation of our manuscript and for their constructive and insightful comments. We have revised the manuscript thoroughly to improve clarity, consistency, and overall quality. All changes have been incorporated into the revised version. Our detailed responses are provided below.

**Reviewer #1:**

For a journal like PLOS ONE, which does not perform copy-editing, clarity and correctness in English are essential. By addressing the following points, the authors can significantly improve the quality of the English prose, making the manuscript more professional, easier to read, and more suitable for publication:

**Response:** We appreciate the reviewer’s emphasis on clarity and correctness in English, which is particularly important for PLOS ONE. The manuscript has been carefully revised to address all the points raised.

**Comment 1.** The phrase "(is for slice thickness of 2 mm)" is grammatically incorrect and awkwardly inserted. It breaks the flow of the sentence. Rephrase for clarity. For example: "As shown in Figure 15 for a slice thickness of 2 mm, the solution stabilized..." or "Figure 15 shows the results for a 2 mm slice thickness, where the solution stabilized...".

**Response:** Thanks. We agree with the reviewer and have revised the sentence to improve grammatical correctness and readability. The phrase has been reworded and smoothly integrated into the sentence.

**Revision made:**
The sentence now reads:

“As shown in Figure 13 for a slice thickness of 2 mm, the solution stabilized..." or "Figure 15 shows the results for a 2 mm slice thickness, where the solution stabilized...”

**Comment 2.** Throughout the manuscript: There are minor inconsistencies, such as the use of "et al" versus "et al." (the latter, with a period, is standard). Also, check for consistent capitalization in titles and headings.

**Response:**
Thank you for pointing this out. We have carefully reviewed the entire manuscript and corrected all instances to ensure consistency.

**Comment 3.** "The theoretical model exhibited a high correlation coefficient (R²) ranging from 0.99 to 0.97, with a MAE between 0.063 and 0.186, and a RMSE ranging from 0.092 to 0.275." The sentence is long and lists many statistics. While grammatically correct, it could be clearer.
Correction (for better readability): "The theoretical model demonstrated a high correlation with experimental data, achieving R² values between 0.97 and 0.99. The model's accuracy was further confirmed by low Mean Absolute Error (MAE) values (0.063–0.186) and Root Mean Square Error (RMSE) values (0.092–0.275)."

**Response:**
We agree with the reviewer’s suggestion and have adopted the revised wording to improve clarity and readability.

**Revision made:**
The sentence has been revised to:

“The theoretical model demonstrated a high correlation with experimental data, achieving R² values between 0.97 and 0.99. The model’s accuracy was further confirmed by low Mean Absolute Error (MAE) values (0.063–0.186) and Root Mean Square Error (RMSE) values (0.092–0.275).”

**Reviewer #2:** The manuscript has been thoroughly and carefully revised according to the comments and is now suitable for acceptance in its current form.

**Response:**
We sincerely thank the reviewer for their positive evaluation and support. We appreciate the time and effort invested in reviewing our work.
